# Male perspectives on intimate partner violence: A qualitative analysis from South Africa

**DOI:** 10.1371/journal.pone.0298198

**Published:** 2024-04-16

**Authors:** Krysta A. Pelowich, Tosin Akibu, Jennifer Pellowski, Abigail Hatcher, Dumisani Rebombo, Nicola Christofides, Karen Hampanda

**Affiliations:** 1 School of Public Health, Brown University, Providence, Rhode Island, United States of America; 2 Department of Community Health Sciences, Fielding School of Public Health, University of California, Los Angeles, Los Angeles, California, United States of America; 3 School of Public Health, Faculty of Health Sciences, University of the Witwatersrand, Johannesburg, South Africa; 4 UN Women Nigeria, Abuja, Nigeria; 5 Department of Behavioral and Social Sciences, School of Public Health, Brown University, Providence, Rhode Island, United States of America; 6 Division of Epidemiology and Biostatistics, School of Public Health, University of Cape Town, Cape Town, South Africa; 7 Department of Health Behavior, School of Public Health, University of North Carolina, Chapel Hill, North Carolina, United States of America; 8 Sonke Gender Justice, Johannesburg, South Africa; 9 Center for Global Health, University of Colorado Anschutz Medical Campus, Aurora, Colorado, United States of America; 10 Department of Obstetrics and Gynecology, University of Colorado Anschutz Medical Campus, Aurora, Colorado, United States of America; University of the Witwatersrand, SOUTH AFRICA

## Abstract

**Background:**

Intimate partner violence (IPV) affects one in four women globally and is more commonly enacted by men than women. Rates of IPV in South Africa exceed the global average. Exploring the background and context regarding why men use violence can help future efforts to prevent IPV.

**Methods:**

We explored adult men’s perspectives of IPV, livelihoods, alcohol use, gender beliefs, and childhood exposure to abuse through a secondary analysis of qualitative interviews that were conducted in South Africa. The setting was a peri-urban township characterized by high unemployment, immigration from rural areas, and low service provision. We utilized thematic qualitative analysis that was guided by the social ecological framework.

**Results:**

Of 30 participants, 20 were residents in the neighborhood, 7 were trained community members, and 3 were program staff. Men reported consumption of alcohol and lack of employment as being triggers for IPV and community violence in general. Multiple participants recounted childhood exposure to abuse. These themes, in addition to culturally prescribed gender norms and constructs of manhood, seemed to influence the use of violence.

**Conclusion:**

Interventions aimed at reducing IPV should consider the cultural and social impact on men’s use of IPV in low-resource, high-IPV prevalence settings, such as peri-urban South Africa. This work highlights the persistent need for the implementation of effective primary prevention strategies that address contextual and economic factors in an effort to reduce IPV that is primarily utilized by men directed at women.

## Introduction

South Africa has one of the highest rates of intimate partner violence (IPV) globally [1]. IPV, as defined by the Centers for Disease Control and Prevention (CDC), refers to “physical violence, sexual violence, stalking and psychological aggression, including coercive tactics, by a current or former intimate partner (i.e., spouse, boyfriend/girlfriend, dating partner, or ongoing sexual partner)” [2]. In most heterosexual relationships in South Africa, men are the users (i.e., perpetrators) of violence rather than the ones experiencing it [3]. The impact that IPV has on women and girls in South Africa are numerous. IPV can cause immediate and direct health consequences such as acquisition of sexually transmitted infections (STIs), including HIV [4]. Physical injury is also commonly reported. Other adverse effects of IPV can be seen through diverse mental health outcomes including depression, post-traumatic stress disorder (PTSD), substance use, and diminished self-esteem [5]. If a woman is pregnant at the time of IPV exposure, indirect health issues, such as low birth weight of child and premature birth of a baby are also possible [1]. Death is another potential outcome of IPV. The WHO estimates between 40–70 percent of female murders that occur globally are committed by a husband or boyfriend, often in the context of an abusive relationship [6].

Data collected in South Africa highlights the varying prevalence of IPV. Some of the underlying causes that impact IPV prevalence can be attributed to geographic location, age, gender, and by study [7]. For example, the most recent Demographic and Health Survey reported that over 25 percent of ever-partnered adult women have experienced IPV during their lifetime with 13 percent reported experiencing IPV in the last year [8]. Among non-representative samples, Selin et al. (2019) reported a 37 percent prevalence of IPV in adolescent girls aged 13–20 years living in rural South Africa [9]. Zembe et al. found a much higher prevalence of IPV, 86 percent, among young women aged 16–24 years residing in the urban city of Cape Town, South Africa [10].

While the majority of research that is conducted on IPV–both globally and in South Africa–focuses on women’s perspective, some data have been collected among men. In a study by Dunkle et al., quantitative surveys with 1275 young men in rural South Africa found 31.8 percent of the participants self-reported using violence against their female partner [11]. Of these, the primary type of violence used was solely physical (71.9 percent). The next common form of violence reported was both physical and sexual violence (16.8 percent) [11]. The use of only sexual violence was the least common (11.3 percent). In a study conducted by Jewkes et al. (2008) across three South African districts, men self-reported a prevalence of using physical IPV at 30.7 percent [12]. Within this study, disclosure of men who raped their partner was 29.6 percent [12]. In peri-urban township settings near Johannesburg and eThekwini (Durban), roughly half of men reported using some form of IPV within the last year [12, 13]. Integral to providing effective primary prevention is understanding the male perspective of IPV, particularly in a context where violence is used more than the global average [1].

Despite the high prevalence of men’s use of IPV in South Africa, much less is known about the key factors contributing to male violent behavior in this geographic context. Some men may consider violence as a way to maintain power and dominance over women, and enact hegemonic masculinity [14]. According to the Theory of Gender and Power, social norms can exacerbate men’s use of IPV in settings where male dominance tends to be acceptable [15]. Recent literature on hegemonic masculinity, however, argues that masculinities are configurations of behaviors developed over time and that understanding men’s life histories is critical to interpret the role of masculinity in IPV [16]. Unfortunately, there is a paucity of research establishing which exposures at various levels of the social ecology across the life-course lead to South African men’s use of IPV and men’s interpretation of their experiences. Further, it is rare that interventions are able to effectively reduce the use of IPV utilized by men [17]. The primary aim and objective of this research is to explore adult male perceptions of IPV in a peri-urban township setting in South Africa by conducting a secondary analysis of a pre-collected qualitative data set. This information will be used to further understand why men use violence and provide a framework to developing acceptable, effective interventions. The secondary aim is to explore key aspects of early life trauma and what impact that has on adult relationships.

## Methods

### Data source

The data was collected in 2015 as part of formative research for a community-level intervention that focused on preventing IPV use among adult males. The study was approved through the human research ethics committee (HREC) of the University of Witwatersrand, South Africa (HREC M160361). The interviews helped the research team make important refinements to an existing gender-transformative program developed by Sonke Gender Justice, an organization focused on combatting gender-based violence, HIV/AIDS, and child abuse. Sonke Gender Justice is part of the Violence Prevention Alliance (VPA) which is a network of WHO members, international agencies, and civil society organizations working to prevent violence while increasing awareness of human rights and promoting healthy attitudes towards masculinity [18].

Purposive sampling was completed by two key informants that worked at Sonke Gender Justice and had strong ties to the local community. The key informants identified, approached, and invited men to participate. Individuals that were interested in the study were then contacted by a trained researcher to schedule a time for their interview (n = 30). Inclusion criteria required that men be over 18 to participate so they could legally consent, and they must have been living in the same peri-urban township during 2015.

Twenty of the men were from the general population of community members, 7 were community action team members that were local volunteers who received a stipend from the program and additional training. The final 3 were Sonke Gender Justice staff members who received training, ongoing supervision, and a monthly salary. The three groups of men had homogeneous characteristics and were asked similar semi-structured interviews, therefore, we examined all 30 men’s responses together. This allowed for a larger sample size and breadth of perspectives to be analyzed.

The interviews took place in a peri-urban setting near Johannesburg, South Africa, and focused on the topics of employment, fatherhood, IPV interventions, perceptions of IPV, and gender norms. Written informed consent was obtained from the men who opted to participate. The qualitative researcher conducted the interviews in the participants chosen language of isiZulu or Sesotho and, with permission, audio recorded the conversation using a digital application. A trained transcriptionist translated the interview from isiZulu or Sesotho to English and typed English transcripts verbatim, with italics for important phrases that were spoken in the local language. Interviews took approximately 60 minutes and were held wherever the interviewee was comfortable [19]. This meant that for some participants, interviews were conducted in their homes while for others, they were conducted in a church or community hall as long as privacy was maintained [19].

### Data analysis

To follow best practices in qualitative methodology, the research team thoroughly read and re-read the transcripts to familiarize themselves with the text prior to any coding. After this process, the team began to apply structured coding to the transcripts utilizing Atlas.ti. *A priori* codes were created based off of the parent study’s interview guide and codes that were identified in a previous analysis (see S1 Appendix). This was used as a framework when the team began to read and set up an initial codebook.

To establish interrater reliability, two authors (KP & KH) used the same codebook to independently code the same interviews. They then discussed any discrepancies they found and came to consensus. This was done for 10% of the interviews. After this subset of interviews was coded, any necessary revisions were made, and the final codebook was established. The final codebook contained thematic code names and definitions which the research team used to complete data analysis by applying codes to varying text (see S2 Appendix).

The full analysis team (KP, JP, AH, AK) reviewed the initial results and interrogated the findings, which resulted in additional queries and validity checks of the analyses. Utilizing the final data that had been coded, the analysis team wrote qualitative analytical reports on each major thematic code.

## Results

### Participant characteristics

Participants had been residing in the area between 4 months to 20 years. The majority were either unemployed or self-employed as day laborers. Ages of participants ranged from 18 to 57 years and most individuals lived with a romantic partner. Roughly half of the participants spoke about having children of their own, though we did not collect demographic data on this aspect of their lives. None of the participants were screened or assessed for use of IPV prior to the qualitative interviews.

In the following sections, we present the main themes from the qualitative interviews related to men’s experiences and perceptions of IPV.

#### 1. Alcohol usage is linked to violence

Participants described that the use of violence, including IPV, was often linked to alcohol consumption. It was common for men and women to gather at bars, or shebeens (i.e. local, informal, and unlicensed drinking areas), and begin drinking in the afternoons. As the evening continued, violence in the streets would break out between community members, gang members, and between partners. When one participant stated that “*…people around [here] actually like violence…” (*CAT07) the interviewer asked him what might be the reason. The participant replied, “*we drink too much*” (CAT07).

Another participant described often hearing his neighbors fight and he perceived them as being in an abusive relationship. He explained that when a fight erupted between the couple, he could hear it. When the participant was asked what he thought might cause the fights, he also felt that alcohol played a role.

*“I think sometimes it [use of violence] is alcohol*. *When they are sober they are okay [not using violence]*. *When they start drinking everything goes wrong*.*”* (FORM11)

This connection is further supported by participants’ observations of the lack of violence during the day when people are less likely to be drinking. A conversation between an interviewer and participant illustrated this:

*I*: *I have observed that now in the afternoon we do not hear any cries for help or shouting*

[*due to violence]*.

*R*: *No*, *no cries at all*.

*I*: *Because people are sober*?

*R*: *Exactly*, *because people are sober…*.*when [the clock] strikes 12 at night there will be no peace*. (FORM20)

These three excerpts describe how there is a perceived direct link between alcohol consumption and violence. The violence described by participants included both community violence and IPV.

#### 2. Socioeconomic context of violence

While many participants noted in their interviews that they moved to the peri-urban settlement in hopes of finding an occupation, several were still unemployed. Having no income to support one’s family and living in an impoverished area created significant stress for men. Some participants, such as CM02, described how they perceived the connection between economic hardship and violence:

*“…maybe there is no electricity and there is no water and others—they are not even working—that kind of thing can pile up into stress whereby someone he took his frustration out to his partner because he is not working*, *he can’t afford to buy food*, *the kids don’t have shoes to go to schools*.*”* (CM02)

Alternatively, those that were able to obtain employment and financial means sometimes used their economic status as leverage over their partner, thus creating a situation for violence to start. CAT04 expressed how the male “provider” role, based on the social construction of gender norms, allowed men to provoke fear in their partner and prevent them from reporting IPV to the police as it could inhibit household wellbeing:

“…*men think they have power over a woman … because maybe I’m a breadwinner*. *If you fight me back*, *I will stop buying food*, *I will stop paying rent if I’m renting*… *If my wife wants something*, *I’m the one who’s going to buy*, *so if my wife fights me back*, *she’ll be afraid*. *And if she goes to the police station and reports*, *she will think about if she reports her man … they’re going to arrest him and he’ll lose his job*.*”* (CAT04)

Even though the economic status of participants fluctuated, violence remained a continuous issue. The narratives above describe how both economic hardship and men earning money can be related to their use of IPV.

#### 3. Perception of gender and constrained views around manhood

The participants perceived gender roles in a variety of ways. For some, it meant the differences in household responsibilities. Participant CAT01 explained that men would be made fun of if they were seen “*doing washing and just cooking and cleaning around*” because it was “*women’s work*” (CAT01).

Some of the participants described women as being more passive compared to men when characterizing the perceived behavior differences.

*“The role of a woman in the house is supposed to be submissive*, *that’s all*. *She doesn’t have a say whether in finance or anything in the house—you guys don’t have to share some responsibility in the house*.*”* (CM02)

In this example, the participant illustrates the perceived culturally prescribed power dynamics that differ between men and women. Women are not to question what men are doing with the household decision making. Similarly, some participants discussed gender power dynamics in relation to how they felt women should act in society. CM06 explained,

*“It’s kind of normal for me to be seen smoking and then two minutes it’s a lady smoking*, *it’s kind of weird to people*. *They don’t expect a woman smoking and then if she’s smoking*, *obviously she’s going [to] be called names…It’s also becoming normal for ladies to wear trousers…can’t you see they’re undermining us men*?*”* (CM06)

In this quote, the participant is sharing their perception of gender differences in relation to smoking behavior, as well as clothing. Within the community, smoking is considered a masculine behavior. Additionally, the simple act of a woman wearing trousers, which is perceived as a standard item of clothing for men, begins to encroach on men’s masculinity. When both genders exhibit equal behaviors, it can be perceived as threatening for some men. It is postulated that during social transitions, such as changes in gender roles, IPV may temporarily increase due to men’s backlash against women assuming more equitable roles [20].

To clarify what the male participants perceived as masculine behavior, they were specifically asked to provide examples on what masculinity meant to them. Nearly every participant described masculinity as being synonymous with pride, responsibility, and coming-of-age. Some participants described going through a ceremony within their tribe, during which they became a man. Others described masculinity as providing for their family. FORM14 explained, “*in this area*, *being a man means having money*, *you cannot live here if you don’t have it*”.

Another form of masculinity was described as asserting sexual dominance. CAT01 stated that masculinity meant having “*one-night stands*,” while CAT02 described it as “*having lots of girlfriends*”. CAT02 further expanded on the meaning.

*“Just to brag that I’ve slept with–let me say–[I made 15 kids with] 15 ladies this weekend*.*”* (CAT02)

This example of young men in the community boasting about having multiple sexual partners was not singular. It was a common theme among the men due to hegemonic masculinity fostered by the perceived power and expected subordination of women. Women’s subordination can be additionally justified in a marriage if the man paid “lobola” (i.e., bride price paid by the man to the woman’s family). Gender norms, combined with the lobola custom, can have implications on men’s justification for the use of IPV, as illustrated by the following quote:

*“I think the men*, *that is why they used to abuse their wives*. *They think they are strong or they think no one can tell them what is right*, *so that is why they are abusing the women*. *They have that thing…*.*so he has to do that because he paid lobola [bride price] and everything so I have to abuse…it is good I can beat her…because I paid for her*, *it’s like having a car*.*”* (CM01)

The quote above highlights how hegemonic masculinity is exerted through violence and control. Men’s perspective that violence and control over women are acceptable behaviors was further exacerbated by paying lobola, which gave them a sense of ownership over women.

#### 4. Familial violence across generations

A final theme that was salient in the interviews was generational violence. For example, some participants described experiencing violence in their childhood from their own father, referencing “the stick” as a tool in which they were struck with. FORM05 explained that during his own childhood, “*the stick was highly functional*. *If you made a mistake*, *they [parents] did not talk too much*, *you knew you were going to be beaten*. *Those were the ways in those days*. *Even at school*, *if you did not perform well*, *you were beaten so you could go and read*.” Several participants described the continued use of violence as a method to teach and discipline their own children.

*“He [son] knows*, *when he is naughty*, *he knows*. *When he gets naughty*, *I spank him with a belt just a little bit*. *I then talk to him and tell him that what he did was wrong and that I did not like it*. *I also warn him that if he repeats what he did I will spank him again*.*”* (FORM02)

Alternatively, other participants in our sample described how violence exposure in their childhood made them hesitant to use the same force with their own children. When asked about the differences between their own childhood and how they parent currently, one participant responded that he used words rather than physical violence to teach his children, which was in sharp contrast to his experience as a child:

*“It’s not the same*, *I grew up in a household where I would occasionally get spanked*. *I think long and hard before spanking my kids because children of today are very sensitive and will take physical discipline as not being loved by their parents*. *I would rather sit down and speak to my children with their mother*. *We used to be hit all the time*.*”* (FORM18)

There is a difference in how the participants described using violence towards their partner as opposed to using it towards their children. Some participants reflected on their childhood experiences and chose to use different methods for disciplining their own children, yet it is unclear why violence used by participants towards their partner continued. While participants may have intended to create a better future for their children by the reduction in violence directly used on them, the imprinting that occurred to their children when violence took place between parents was just as significant.

*“This thing*, *it comes from generation from generation*, *when we say I might want to do what my dad does at home*, *you know*, *problems start at home*. *Because*, *you find out that both parents are drinking and then they fight*, *you know*, *so they keep close up*, *seeing everything*, *experiencing this*. *Now you just want to show off*. *My dad beats my mom like this and that*, *my dad smokes in front of me*, *so sometimes*, *I even drink with him*.*”* (CAT02)

In the above quote, the participant acknowledged the intergenerational transmission of both violence and alcohol use. Engaging in similar behaviors as male role models in the home (e.g., fathers), even if problematic, may allow boys social opportunities to connect with their fathers. This quote illustrates the tension between healthful behaviors and finding forms of connectedness, particularly as a child in constrained environments.

## Discussion

This qualitative study investigated the complexities behind IPV from the male perspective in a low resource setting with high community violence and IPV in South Africa. While there is a vast literature on gender power dynamics and IPV, there is a gap in research on men’s perceptions of factors that influence the use of IPV, particularly in global settings [21]. At each level of the social ecology (sociocultural, economic, interpersonal and familial, and individual), male participants in the present study provided examples of influential factors connected to the use of IPV against women, supporting prior research on the usefulness of applying the social ecological model to the study of IPV [12, 13, 22]. Unfortunately, our participants emphasized the high levels of various types of violence in the community, which created an atmosphere where violence, including IPV, became normalized.

This study revealed the importance of the life course perspective and social learning on adult men’s perceptions and behaviors, including the use of IPV. The findings of the present study are in line with the Intergenerational Transmission of Violence Theory and social learning theory, which both posit that individuals will imitate the behaviors of influential role models, including interpersonal violence they may have witnessed in their household as children [23, 24]. Our participants explicitly described how men in this setting often engage in behaviors that they observed from male role models (“[I] do what my dad does”). This included the use of violence as a form of discipline for children, the use of IPV against women, and substance use. Prior research from multiple global settings has similarly reported that experiencing childhood abuse and growing up with domestic violence are risk factors for IPV [25, 26]. Our findings highlight that the men in the present study are consciously aware of this connection and viewed the violence they witnessed or experienced as problematic. Despite this recognition, none of the participants described explicit attempts to stop the intergenerational cycle of IPV against women. Conversely, some participants did describe active attempts to stop the cycle of physical violence against children (e.g., talking instead of spanking). More research is needed to explore how men in different contexts understand the consequences of experiencing violence as a child (direct victimization) *compared to* witnessing violence against women (indirect/vicarious victimization), which could be leveraged in future prevention efforts.

Our participants continuously highlighted the complexities of how Black South African men are expected to attain dominant cultural standards of masculinity within the constraints of post-colonial/post-apartheid economic marginalization [27, 28]. In our interviews, men consistently endorsed the notion that to fulfil masculine gender norms, they are expected to work outside the home and be the main financial providers of the family. Most participants described coming to the peri-urban township setting in hopes of finding employment, yet they remained jobless. Finding employment was extremely difficult in this setting due to high levels of unemployment and economic precarity. When men could not adequately fulfill their expected masculine role as the breadwinner, men often experienced a high level of stress, which was subsequently exhibited in the form of violence, including IPV. Prior research has also noted that income loss and an increased time at home are both drivers of IPV [29]. We concur with other scholars who argue that IPV researchers need to consider how constructions of masculinity in Sub-Saharan African settings are entangled in complex heteropatriarchal-capitalist configurations based on a unique historical political economy that has disenfranchised certain groups of men [27, 30].

Yet, our participants reported a somewhat contradictory observation that men’s greater access to economic resources compared to women, when combined with gender norms legitimizing violence, can also exacerbate IPV. This finding supports prior research indicating that in societies where there is unequal access to economic or political resources by gender, the likelihood of IPV against women increases [31, 32]. In our interviews, participants described that when men were successfully able to fulfil their role as the financial provider, women could be in a situation where they are forced to endure IPV because of their economic dependence on the male partner. Prior research has similarly reported that when women are dependent on men for economic and social capital, a potential consequence can be the acceptance of IPV and staying in violent relationships [33]. While some gender norms differed by participant, men typically emphasized hegemonic masculine ideals, including entitlement over women [34]. Further, within the context of the study location, it is not uncommon for lobola, or bride price, to be paid from the man to the bride’s family prior to marriage, which may additionally increase men’s level of entitlement and acceptance of IPV because they “own women” [35].

Alcohol use, the most prominent theme we discovered, has been associated with violent crimes, anger and IPV [36]. This, however, is not a new finding. Researchers have been looking for a causal link between alcohol and violence for over thirty years [37]. While it is hard to pinpoint the mechanism, it is clear that excessive alcohol exacerbates violence. Further, the primary form of social networking in the peri-urban township took place at bars, or shebeens, where alcohol consumption occured. Participants described IPV to either start at the bar, if the male brought his female partner with him, or it began when he went back home.

Current programs, such as Sonke Gender Justice, exist to help implement gender transformative interventions. Sonke Gender Justice holds trainings that aim to reduce gender inequality, and engage men in IPV prevention efforts [38]. Equimundo, founded in Brazil, is another organization that works globally to engage men in gender equality. Their programs include breaking down traditional gender norms, empowering women in the economy, and combating homophobia [39]. Other gender transformative programs include the qualitative evaluation by Gibbs et al. on the Stepping Stone’s intervention, which worked to transform men’s gender norms, build gender equality, and enhance men’s economic opportunities [40].

The use of qualitative research in this topic area is important because it allows for the community members to describe their own experiences with IPV and greater clarity surrounding the topic can be acquired. This research helps to fill the gap as it highlights areas in which our attention could be focused on, particularly alcohol use, economic opportunities for men, gender norms, and breaking the cycle for family violence. The findings from this study will be used in the future as formative work for creating culturally appropriate primary prevention strategies. Further research should focus on developing and implementing acceptable and effective intervention approaches to prevent men from ever using IPV. In addition to the more common interventions aimed at assisting women experiencing IPV, work should also focus on assisting men to cease their use of IPV.

### Limitations

A few limitations of this study should be highlighted. This study utilized secondary data analysis and thus, interview questions were not created a priori with regards to this specific research question and the Theory of Gender and Power was not used to frame the initial interview questions. This posed some challenges during analysis as we could not probe further to illustrate the associations of identified perceptions to use of violence. With this particular data set, we aimed to explore topics related to gender norms and early life experiences but also found significant information regarding alcohol use and employment status. Due to the qualitative nature and in person interviews, social desirability bias and recall bias were also possible. While we utilized team coding to strengthen the findings, it is important to note the positionality of the primary researcher. The secondary data analysis was led by a white, Global North female with limited time in the community of focus. Co-authors who led primary data collection, ethnographic, and trial data collection over the course of several years offered contextual insights to overcome this limitation. Finally, few demographic information was collected on the participants which limited our ability to comprehensively understand the sample population and be able to compare participants within the sample.

## Conclusion

In this study with 30 men living in a peri-urban township of South Africa, we exploried their perceptions of IPV. The main themes were that gender power dynamics, early life trauma, alcohol consumption, and lack of economic opportunities were perceived to fuel IPV enacted by men towards women. This highlighted the various levels of the social ecology that need to be addressed in order to prevent men’s use of IPV. Integral to providing effective primary prevention is understanding the male perspective of IPV, particularly in a context where violence is used more than the global average. While IPV interventions overall are lacking, those that exist primarily focus on limiting the harmful effects that women experience after an IPV event rather than promoting primary or secondary prevention strategies among those who use violence.

## Supporting information

S1 Appendix *A priori* themes. (DOCX)

S2 Appendix Codebook and definitions. (DOCX)
