# Supplementary material for: Male perspectives on intimate partner violence: A qualitative analysis from South Africa
Source: PLoS One. 2024 Apr 16;19(4):e0298198. doi: 10.1371/journal.pone.0298198 (PMC11020850; doi:10.1371/journal.pone.0298198)
Supplement: S1 Appendix — (DOCX) [file pone.0298198.s001.docx]

Appendix A. *A priori* themes

| ***A priori* theme** |  |  |
| --- | --- | --- |
| Alcohol abuse | Fatherhood | Poor communication |
| Background | Gender norms | Poor economic status |
| Child neglect | Infidelity | Poor self-worth |
| Drug abuse | Lack of trust |  |
| Employment | Patriarchal culture group |  |
| Environmental Factors | Peer groups |  |
