# Supplementary material for: Male perspectives on intimate partner violence: A qualitative analysis from South Africa
Source: PLoS One. 2024 Apr 16;19(4):e0298198. doi: 10.1371/journal.pone.0298198 (PMC11020850; doi:10.1371/journal.pone.0298198)
Supplement: S2 Appendix — (DOCX) [file pone.0298198.s002.docx]

Appendix B: Codebook and definitions

| Code | Definition |
| --- | --- |
| Alcohol/shebeens | This code refers to alcohol use and drinking at local bars |
| Communication |  |
| Drug use/smoking |  |
| Employment |  |
| Hope |  |
| Housing/infrastructure |  |
| Important |  |
| Inaction |  |
| Involvement with project |  |
| Perception of “masculinity” |  |
| Perception of community violence | This code is to describe the violence experienced within the community, not specific to IPV |
| Perception of Diepsloot |  |
| Perception of family violence |  |
| Perception of gang activity |  |
| Perception of gender |  |
| Perception of violence and alcohol | This code is used to describe the link between violence and alcohol. |
| Perception of violence and money | This code is used to describe the link between violence and money. |
| Perception of youth |  |
| Police/jail | This code is used to describe the legal infrastructure related to violence |
| Pre-Diepsloot life |  |
| Rape |  |
| Reason to change |  |
| Relationships | This code is used for describing relationships between family members |
| VAW |  |
| Violence; personally experienced |  |
